# Supplementary material for: apterous A specifies dorsal wing patterns and sexual traits in butterflies
Source: Proc Biol Sci. 2018 Feb 21;285(1873):20172685. doi: 10.1098/rspb.2017.2685 (PMC5832707; doi:10.1098/rspb.2017.2685)
Supplement: Supplementary Materials [file rspb20172685supp1.pdf]

Supplementary Materials for

apterous A specifies dorsal wing patterns and sexual traits in butterflies

Anupama Prakash* and Antónia Monteiro*

correspondence to: anupama@u.nus.edu or antonia.monteiro@nus.edu.sg

DOI: 10.1098/rspb.2017.2685

This file includes:

Supplementary Figures:

- Figure S1: *ap* mRNA localization in developing wing discs of *Bicyclus anynana*
- Figure S2: CRISPR/Cas9 mosaic wing pattern phenotypes of *apB* knockout
- Figure S3: A catalog of the different types of CRISPR/Cas9 mosaic wing pattern phenotypes of *apA* homeodomain knockout
- Figure S4: A catalog of the different types of CRISPR/Cas9 mosaic wing pattern phenotypes of *apA* LIM domain knockout
- Figure S5: A catalog of the different types of CRISPR/Cas9 mosaic wing pattern phenotypes of *apB* LIM domain knockout

Supplementary Tables:

- Table S1: List of primers and guide RNA sequences used in this study
- Table S2: CRISPR/Cas9 injection concentrations and mutation frequencies
- Table S3: Results from a second set of injections with guides and Cas9 mRNA and Cas9 mRNA alone (Control) to test whether presence of each of the guides impacts hatching ratios.

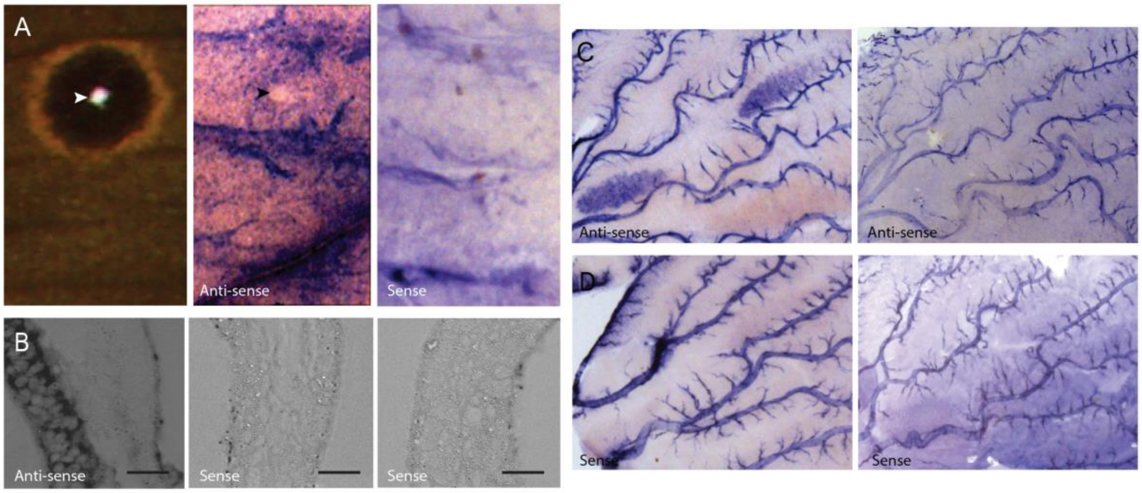

Figure S1: ***ap* mRNA localization in developing wing discs of *Bicyclus anynana*** A) *apA* mRNA localization (middle) in wildtype 5th larval instar wing discs with control (right). There is an absence of *apA* expression in future dorsal eyespot centers (arrowhead). Corresponding adult wing is shown (left). B) Cross-sectional view of a developing wing disc showing dorsal-specific *apB* expression (left). No staining is seen with control probes for *apB* (middle) and *apA* (right). Scale bar is 20µm C) Male (left) and female (right) hindwing discs (28 hours after pupation) showing *apB* mRNA up-regulation in the hair-pencil regions only in males. D) Controls for *apB* (left) and *apA* (right) expression in male wings show no staining in the corresponding regions.

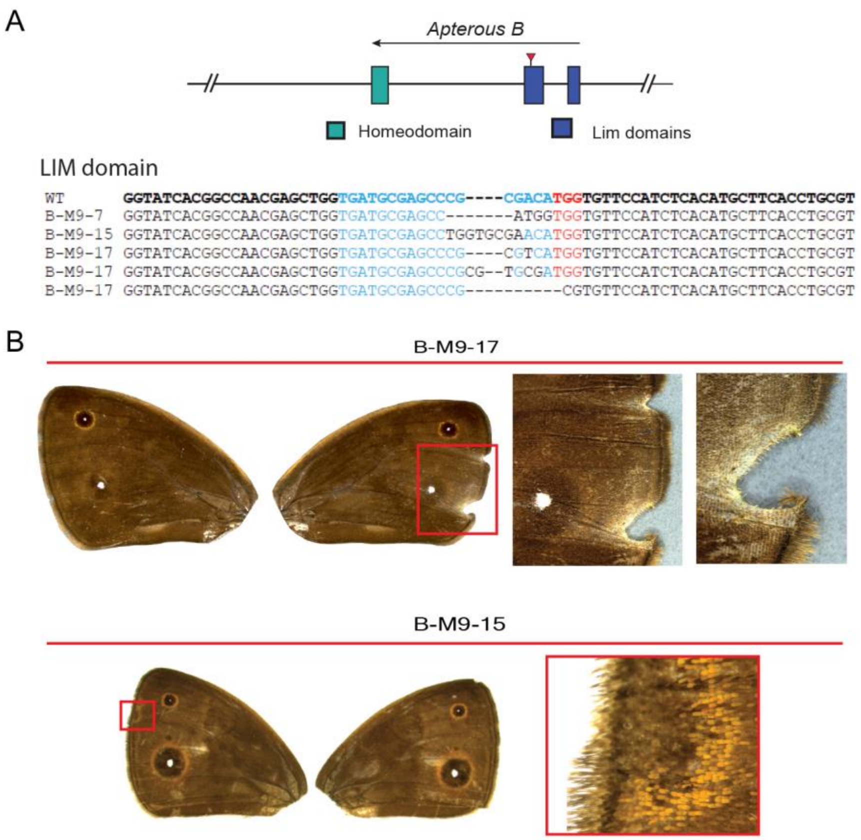

Figure S2: **CRISPR/Cas9 mosaic wing pattern phenotypes of *apB* knockout** A) Top: Region of the *apB* gene in *B. anynana* targeted using the CRISPR/Cas9 system Bottom: Sequences of the LIM domain region of mutant individuals compared with the wildtype sequence in bold. Blue is the region targeted and the PAM sequence is in red. Deletions are indicated with '-'. B) CRISPR/Cas9 *apB* mosaic phenotypes of *B. anynana*. B-M9-17: The forewings of a mutant individual showing differences in shape and marginal defects of the right wing as compared to the left. The boxed area is expanded to the right. B-M9-15: Mutant with wing pattern changes that do not correspond to mosaic ventral patterns, but appear to indicate disruptions to wing margin development. Boxed area expanded to the right.

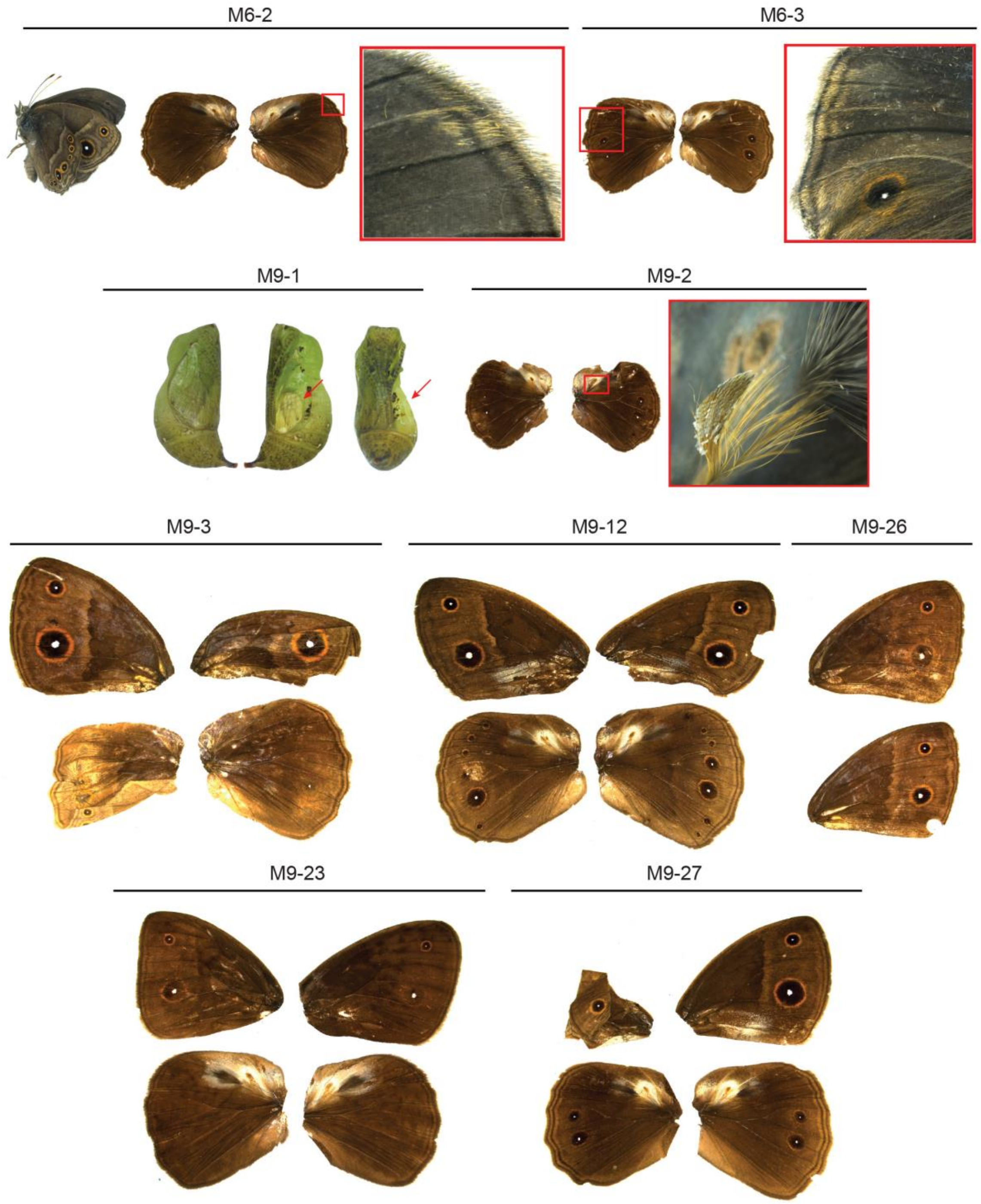

Figure S3: **A catalog of the different types of CRISPR/Cas9 mosaic wing pattern phenotypes of *apA* homeodomain knockout**

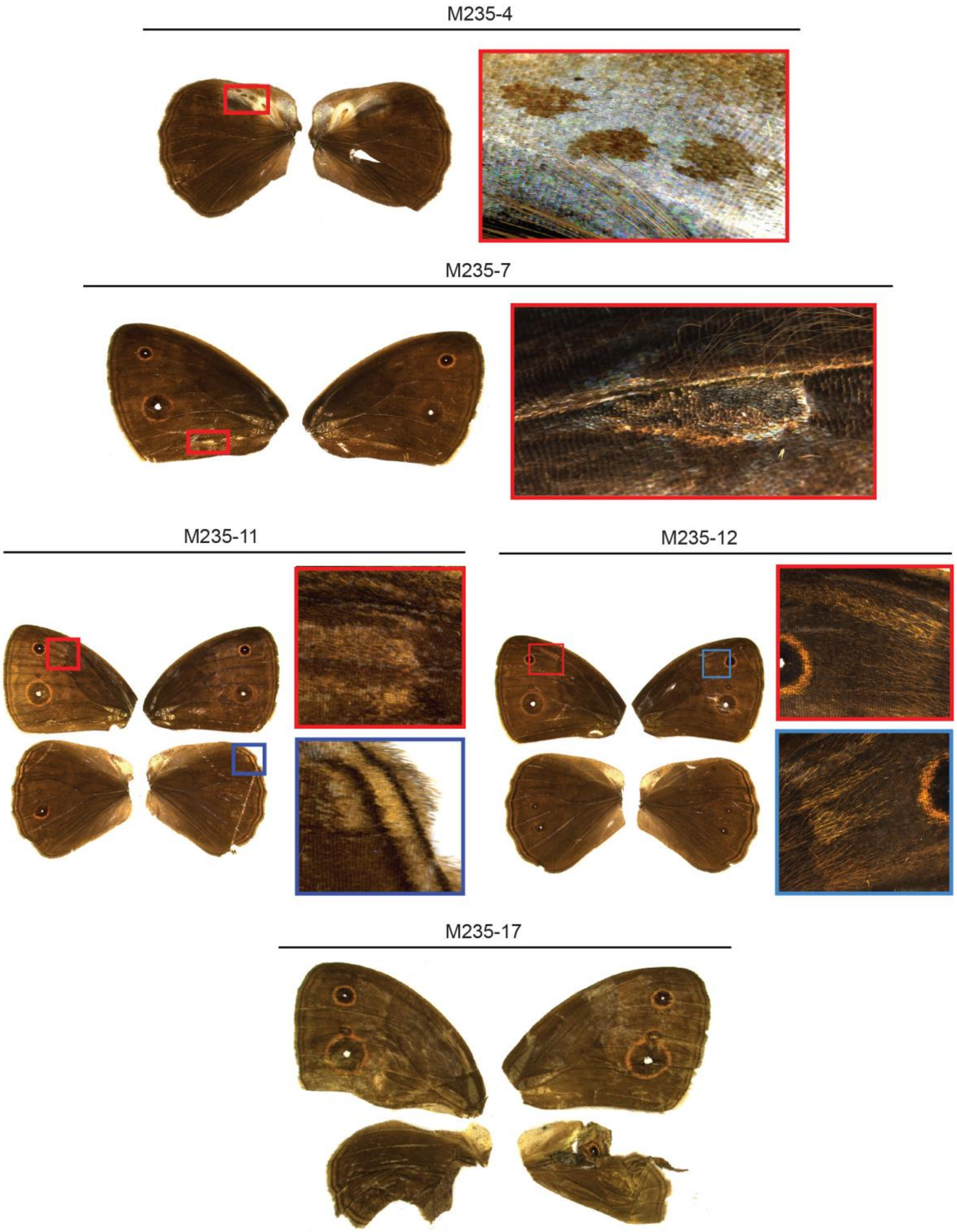

Figure S4: **A catalog of the different types of CRISPR/Cas9 mosaic wing pattern phenotypes of *apA* LIM domain knockout**

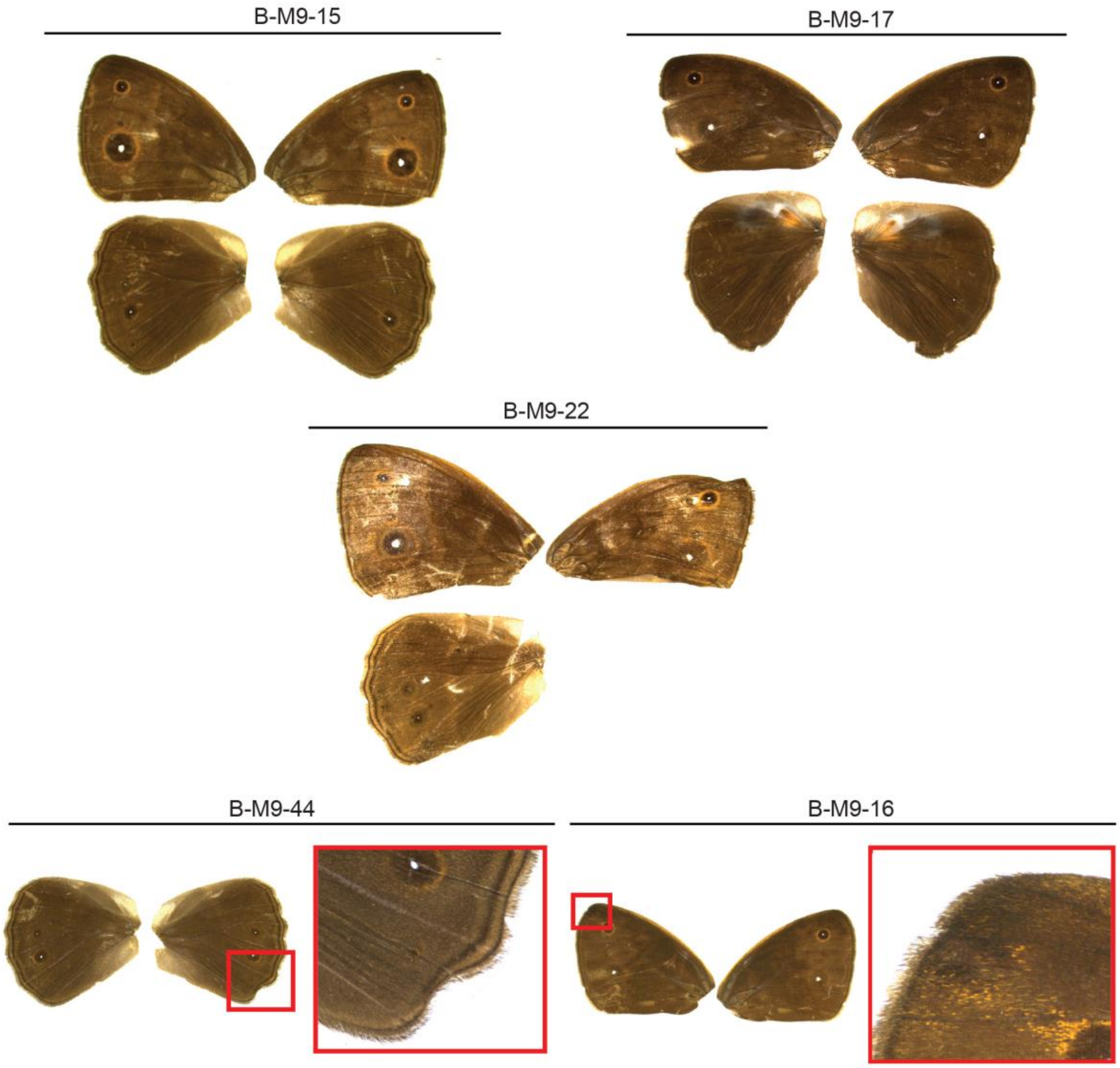

Figure S5: **A catalog of the different types of CRISPR/Cas9 mosaic wing pattern phenotypes of *apB* LIM domain knockout**

Table S1: List of primers and guide RNA sequences used in this study

Gene	Primer Name	Primer Sequence
Apterous A (*ApA*)	AM 31<br>AM 32	Forward 5’ CGGGAGGCCTGTCTTCTGGC 3’<br>Reverse 5’ CGTCGGAGCTGGTGATGAGGG 3’
Apterous B (*ApB*)	AM 136<br>AM 137	Forward 5’ CGAACAGTTGAATGCGTATTG 3’<br>Reverse 5’ GGCCACTTTTCTCTTTCTTGG 3’
ApA Homeodomain CRISPR Guide	AM 158	5’GAAATTAATACGACTCACTATAGGAGCTGGTGATGCTTGAAGCGTTTTAGAGCTAGAAATAGC 3’
ApA LIM domain CRISPR guide	AM 235	5’GAAATTAATACGACTCACTATAGGAGAAACAGTGCACATGAAACACGTTTTAGAGCTAGAAATAGC3’
ApB LIM domain CRISPR guide	AM 145	5’GAAATTAATACGACTCACTATAGGTGATGCGAGCCCGCGACAGTTTTAGAGCTAGAAATAGC3’
ApA Homeodomain Genotyping	AM 194<br>AM 167	Forward 5’ CATTTTTGCGACACGAGACGTC 3’<br>Reverse 5’ CTAACTGTCTCGACTATATG 3’
ApA LIM domain CRISPR Genotyping	AM 257<br>AM 258	Forward 5’ GTACAGTAATTAGTTCATCAAAC 3’<br>Reverse 5’ CTTTTCAGTTGTGTGCATTTTAAG 3’
ApB LIM domain CRISPR Genotyping	AM 385<br>AM 386	Forward 5’ CACTAGATTAGCCTAAGGTC 3’<br>Reverse 5’ CTGTTTTGTAGGAGAAATATGG 3’

Table S2: CRISPR/Cas9 injection concentrations and mutation frequencies

Guide	Guide RNA Conc (ng/ul)	Cas9 mRNA Conc (ng/ul)	Eggs injected	Eggs hatched	Hatch ratio	Total adults	Mutant phenotypes
ApA Homeodomain	360	600	631	55	8.7%	9	3 (33%)
	450	900	882	89	10%	35	9 (25.7%)*
ApA LIM Domain	400	900	266	n.a	n.a	17	6 (35.2%)
ApB LIM Domain	400	900	228	75	32.89%	45	6 (13.3%)

* 4 of the 9 mutant individuals were pupae with wings missing from one side as shown in SFigure 3

Table S3: Results from a second set of injections with guides and Cas9 mRNA and Cas9 mRNA alone (Control) to test whether presence of each of the guides impacts hatching ratios. None of the adults that resulted from this experiment showed mutant phenotypes.

Guide	Guide RNA Conc (ng/ul)	Cas9 mRNA Conc (ng/ul)	Eggs injected	Eggs hatched	Hatch ratio
Control	-	900	103	53	51.4%
ApA Homeodomain	400	900	113	75	66.3%
ApA LIM Domain	400	900	108	51	47.2%
ApB LIM Domain	400	900	104	53	50.9%
